# Identification and validation of regulatory SNPs that modulate transcription factor chromatin binding and gene expression in prostate cancer

**DOI:** 10.18632/oncotarget.10520

**Published:** 2016-07-09

**Authors:** Hong-Jian Jin, Segun Jung, Auditi R. DebRoy, Ramana V. Davuluri

**Affiliations:** ^1^ Division of Health and Biomedical Informatics, Department of Preventive Medicine, Northwestern University Feinberg School of Medicine, Chicago, IL 60611, USA

**Keywords:** SNP, prostate cancer, transcription factor, CRISPR/Cas9, eQTL

## Abstract

Prostate cancer (PCa) is the second most common solid tumor for cancer related deaths in American men. Genome wide association studies (GWAS) have identified single nucleotide polymorphisms (SNPs) associated with the increased risk of PCa. Because most of the susceptibility SNPs are located in noncoding regions, little is known about their functional mechanisms. We hypothesize that functional SNPs reside in cell type-specific regulatory elements that mediate the binding of critical transcription factors (TFs), which in turn result in changes in target gene expression. Using PCa-specific functional genomics data, here we identify 38 regulatory candidate SNPs and their target genes in PCa. Through risk analysis by incorporating gene expression and clinical data, we identify 6 target genes (ZG16B, ANKRD5, RERE, FAM96B, NAALADL2 and GTPBP10) as significant predictors of PCa biochemical recurrence. In addition, 5 SNPs (rs2659051, rs10936845, rs9925556, rs6057110 and rs2742624) are selected for experimental validation using Chromatin immunoprecipitation (ChIP), dual-luciferase reporter assay in LNCaP cells, showing allele-specific enhancer activity. Furthermore, we delete the rs2742624-containing region using CRISPR/Cas9 genome editing and observe the drastic downregulation of its target gene UPK3A. Taken together, our results illustrate that this new methodology can be applied to identify regulatory SNPs and their target genes that likely impact PCa risk. We suggest that similar studies can be performed to characterize regulatory variants in other diseases.

## INTRODUCTION

Prostate cancer (PCa) is the second most common cause of cancer deaths in American men [1]. It is estimated that 220,800 men will be diagnosed and 27,540 men will die of PCa in 2015. Despite its prevalence and lethality, there are still many clinical challenges associated with PCa diagnosis and treatment. Single nucleotide polymorphisms (SNPs) are known to underlie differences in our susceptibility to diseases, and it has also attracted tremendous interest as potential biomarkers for PCa diagnostics and risk prediction in recent years. To date, common genetic variants in more than 70 genomic loci have been clearly associated to PCa by recent genome-wide association studies (GWAS), explaining approximately 30% of the familial risk for this disease [2]. Since the majority of these loci are situated within intergenic or intronic regions, a mechanistic understanding of how they contribute to phenotypes is lacking. Recent understanding of the control of gene expression have emerged as a key tool for connecting DNA sequence variation to phenotypes [3]. It is now clear that regulatory variants can influence the individual steps of gene expression including transcription factor binding [4, 5], chromatin accessibility [6], histone modification [7], DNA methylation [8–10], alternative splicing [11] and so on.

With the advent of high-throughput genomic technologies, genome wide mapping of functional elements became easily feasible. For example, DNase-seq [12] and FAIRE-seq [13] allow us to define DNase I hypersensitive sites (DHS), nucleosome-free regions such as regulatory promoters and enhancers. While chromatin immunoprecipitation assays followed by sequencing (ChIP- seq) can be used to generate high-resolution profiles of histone modifications and transcription factor binding sites (TFBS) [14]. H3K4me1/ H3K4me3 are associated with enhancer/promoter positions respectively and H3K27ac reflects active utilization of these regions [15, 16]. Recent ChIP-seq studies have carefully identified enhancer elements bound by prostate-specific transcription factors such as AR, FOXA1, NKX3-1, GATA2 and HOXB13, revealing the cooperative regulatory network that controls prostate gene expression [17–23]. In addition, The Cancer Genome Atlas (TCGA) has archived a comprehensive molecular characterization of the genetic contributions to PCa. Multiple data types including SNP array and RNA-seq, performed on the same set of patient samples, have been available. These datasets offer unprecedented opportunities for identifying sequence variants that may impact factor binding and gene regulation and thus contribute to this disease.

In addition, various approaches have been developed to identify sequence variants that likely play important biological roles. Gene expression quantitative trait loci (eQTLs) have been widely used to identify the genetic variants impacting gene expression levels [24]. The RegulomeDB tool annotates SNPs with a combination of DHS, TFBS predictions, eQTLs and enhancer information [25]. HaploReg explores annotations of non-coding variants through chromatin states, motif instances and eQTLs from the Genotype-Tissue Expression (GTEx) browser [26]. FunciSNP incorporated ENCODE datasets to annotate putative functional variants in GWAS analysis, providing a comprehensive annotation of the 77 known PCa-risk loci [27, 28]. However, these methods utilize all ENCODE data or all known TF motifs. In reality, distinct cell types are maintained largely through the cell-type specific binding of TFs, and the presence of a motif does not necessarily imply a real TF binding in a different cell type. And eQTL data for prostate is unavailable in the GTEx project [29]. However, both GWAS and eQTL point to regions of linkage disequilibrium (LD) rather than to individual SNPs. It's therefore necessary to identify individual SNPs that overlap regulatory elements.

Using epigenetic marks and open chromatin regions obtained from PCa cells, we identify SNPs that are located in prostate-specific regulatory elements. Through eQTL and motif affinity analysis, we capture a subset of regulatory SNPs that map within a canonical TF binding motif and potentially affect TF genomic occupancy. Using chromatin immunoprecipitation (ChIP) and dual-luciferase reporter assays, we tested several candidate SNPs and confirmed allele-specific TF binding and enhancer activity. Furthermore we deleted one polymorphic enhancer by CRISPR/Cas9 genome editing technology, resulting in altered expression of its target gene. Our studies suggest that this methodology could be used to systematically identify regulatory SNPs in a tissue/disease specific manner and precise deletion of individual enhancers could help to determine their functional significance.

## RESULTS

### Identification of regulatory SNPs and associated genes in PCa

**Figure 1:**
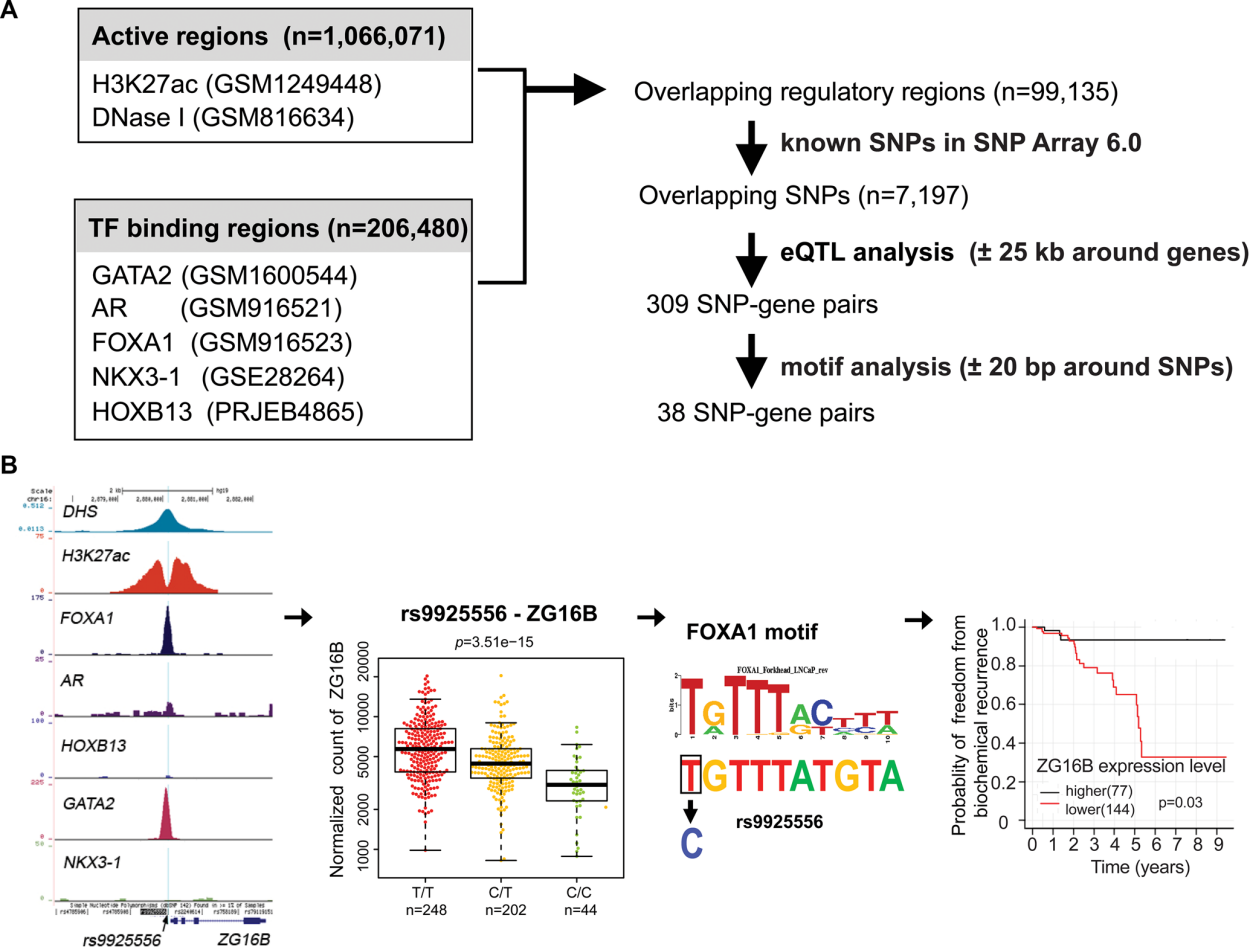
Identification of potential regulatory SNPs for PCa (**A**) Resources, logical workflow, computational processing steps are summarized. First, putative regulatory regions were defined as those bound by one or more TFs and within open chromatin or active histone marks(H3K27ac) in PCa cells (Supplementary Table S4). Considering availability of SNP genotype data, we only chose SNPs that are included in Affymetrix Genome-Wide SNP Array 6.0 platform in TCGA-PRAD studies and reside in our regulatory regions (Supplementary Table S5). The numbers of regulatory SNPs identified by eQTL and further motif analysis are indicated and the lists of candidates are shown in Supplementary Table S6 and Supplementary Table S1 respectively. Furthermore, we performed risk analysis based on gene expression. And several candidate SNPs can be selected for experimental validation. (**B**) shown key steps and a representation of SNP-gene candidates in our analysis. Rs9925556, located in a DHS and H3K272ac marked region, is bound by FOXA1 and GATA2. This SNP is highly associated the expression level of ZG16B gene. Motif analysis revealed that rs9925556 dramatically affects the FOXA1 binding motif. In TCGA PCa dataset, patients with low ZG16B transcriptional activity had shorter BCR-free survival.

The flow diagram presented in Figure 1A depicts the strategy of the systematic processing to acquire, integrate, filter and analyze existing data to identify putative regulatory SNPs in PCa. To obtain PCa-specific regulatory regions, we first combined DNase I hypersensitivity sites and H3K27ac ChIP-seq peaks in PCa LNCaP cells, resulting in over one million regions that represent accessible chromatin regions or active enhancers. Then we retrieved ChIP-seq data sets for AR, FOXA1, GATA2 and NKX3-1 from LNCaP cells and HOXB13 from VCaP cells, generating a set of TF binding regions (*n* = 206,480) with one or more TF binding. Overlapping these TF binding sites with the open chromatin or active enhancer regions, we generated a non-redundant collection of PCa-specific regulatory regions (*n* = 99,135). Through overlapping with genomic positions of SNPs in Affymetrix Genome-Wide SNP Array 6.0 platform used in TCGA PCa project, we found that 7,197 SNPs are located in these PCa-specific regulatory regions.

Next, we analyzed these SNPs to identify potential eQTLs and their target genes using RNA-seq and SNP array data available in the TCGA PCa project. Our previous study and others found that genes located within 25 kb of an AR binding site were the most significantly enriched for androgen-regulated genes in PCa; larger genomic windows could include a greater proportion of false positives [18, 33]. Moreover it's been reported that FOXA1, GATA2, NKX3-1 and HOXB13 can interact with AR and play essential roles in facilitating AR genomic binding and androgen-responsive gene expression [20, 34–36]. The data from the ENCODE consortium estimated that about 47% of the distal regulatory elements have interactions with the nearest expressed transcription start site (TSS) [37]. These analyses suggested that examining the nearby genes within ± 25 kb of SNPs may produce a relatively short but reasonable list of genes potentially regulated by eQTLs within PCa-specific enhancers. Through the eQTL analysis, we identified 309 SNP-gene pairs where each SNP is significantly associated with its nearby gene expression.

To focus on regulatory SNPs that affect TF binding affinity and mediate gene regulation, we particularly excluded SNPs that merely fall within TF binding regions, but locate outside of a canonical DNA binding motif. According to recent advances of gene regulation, only a few of master TFs dominate control of tissue-specific gene expression programs [38]. Furthermore differential TF binding could direct differential histone modifications, DNA methylation and mRNA levels [10]. Thus sequence variation in these tissue-specific enhancers could misregulate gene expression tightly linked to disease [3]. Specifically, we performed a motif analysis using the selected binding motifs of AR FOXA1, GATA2, NKX3-1 or HOXB13. We found 38 SNPs that can cause motif changes for these 5 TFs. 84% of regulatory SNPs (32 of 38) reside at non-coding/distal regulatory region linked to a nearby target gene (Supplementary Table S1). Only 6 regulatory SNPs are located in the gene promoter region that refers to −2kb upstream to 2kb downstream of the TSS [39]. Interestingly, one PCa GWAS SNP rs339331, known to influence its target gene (RFX6) expression by modulating HOXB13 chromatin binding [21], was rediscovered by our analysis. 8 of 38 SNP-gene pairs were identified as eQTLs in the Genotype-Tissue Expression (GTEx) database (www.gtexportal.org), which were considered as genetic changes associated with common human diseases [29]. For example, the gene expression of UPK3A, a promising urinary biomarker for bladder cancer [40], is highly correlated with SNP rs2742624.

### Expression analysis of regulatory SNP-associated genes

We next examined the transcription level of SNP-associated genes using RNA-seq data for 497 PCa samples and 52 normal samples from the TCGA data to determine whether any of these 38 genes are differentially expressed (absolute fold change > 1.5-fold change and *P*-value < 0.01). Seven genes display robust changes including FMO4, EFHD1, MLPH, GIPC2, NAALADL2, KLK15 and LYVE1 (Supplementary Table S2). Of these, EFHD1, MLPH, NAALADL2 and KLK15 are significantly upregulated while others are downregulated in prostate tumors compared to normal samples.

### Clinical impact of regulatory SNP-associated genes on prostate cancer progression

To assess the clinical significance of these SNP-regulated genes, we performed Kaplan-Meier analysis and examined the association of gene expression with clinical variables in a collection of 295 PCa samples from TCGA. We found that the expression of 6 genes—ZG16B, ANKEF1, RERE, FAM96B, NAALADL2 and GTPBP10—showed a strong correlation with frequency of biochemical recurrence (*P* < 0.05). For instance, PCa patients with higher gene expression levels of *ZG16B* had significantly higher risk of biochemical relapse than those patients with lower levels of *ZG16B* expression (*P* = 0.03). Except for rs339331, our analysis identified additional 12 potential PCa-related regulatory SNPs from which 7 SNPs are associated with differentially expressed genes in tumor vs. normal samples while 6 SNPs are involved in risks of biochemical recurrence (Supplementary Table S1 and S2). For example, ZG16B, known to involve in metastasis in colorectal cancer [41], is potentially affected by rs9925556, and lower expression level of ZG16B that is correlated with shorter BCR-free survival in PCa (Figure 1.B). In addition, NAALADL2, overexpressed in colon cancer and PCa, has been reported to play significant roles in cancer development and progression [42]. We also observed the upregulation of NAALADL2 in PCa comparing to normal or benign samples and patients with high expression level of NAALADL2 correlated with shorter BCR-free survival (Supplementary Figure S1). These results support that our method is feasible in identifying causative genes and corresponding regulatory SNPs on PCa risk.

### Effect of selected regulatory SNPs on transcription factor recruitment and Enhancer activity

**Figure 2:**
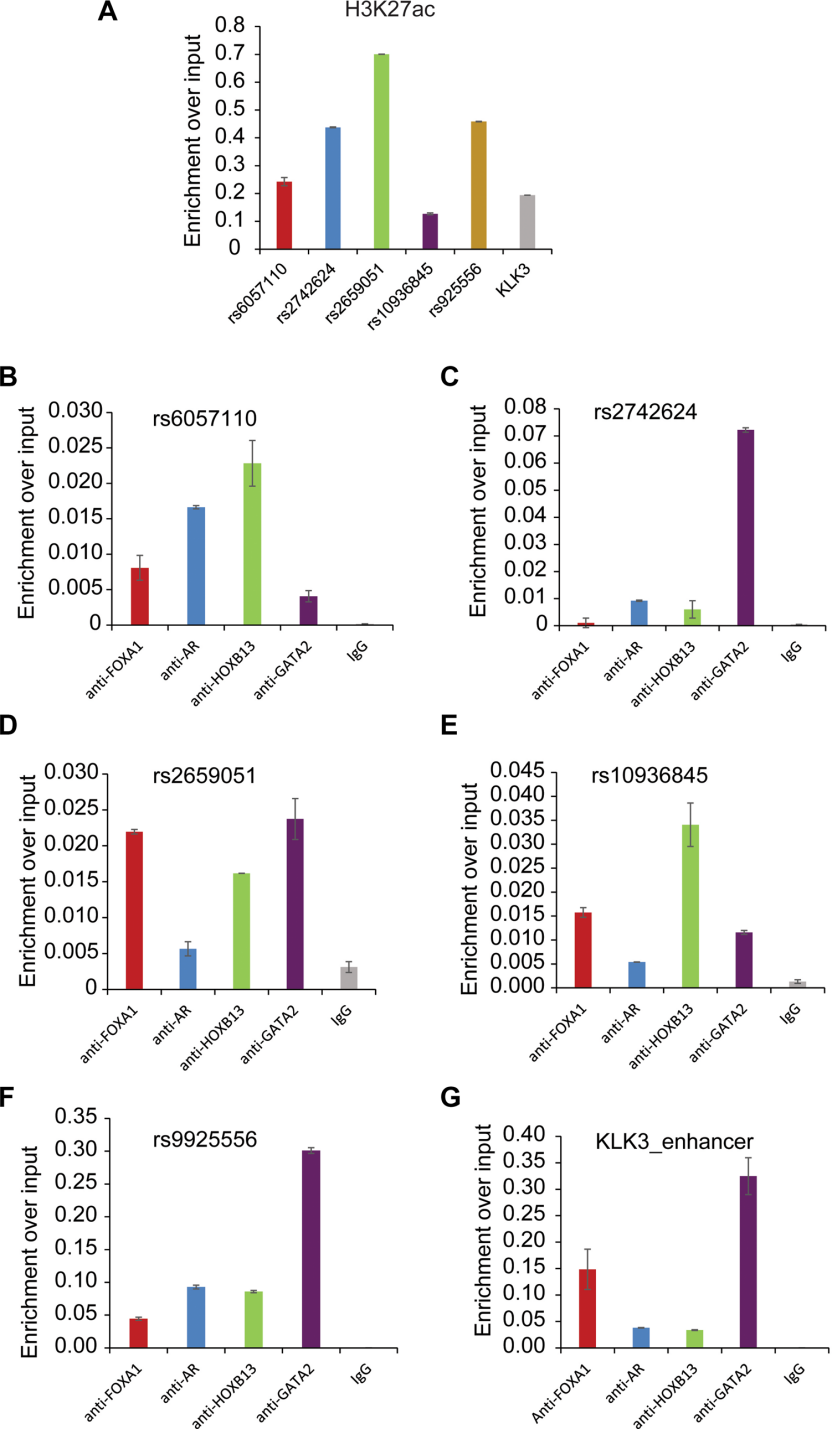
Experimental validation of selected five loci associated with H3K27ac and transcription factor binding H3K27ac, FOXA1, AR, HOXB13, GATA2 ChIP experiments were performed in LNCaP cells and followed by ChIP–qPCR analysis of H3K27ac (**A**) and chromatin occupancy of TFs on 5 selected target regions including rs6057110 (**B**) rs2742624 (**C**) rs2659051 (**D**) rs10936845 (**E**) and rs9925556 (**F**). And KLK3 enhancer locus was tested as a positive control for ChIP-qPCRs.

**Figure 3:**
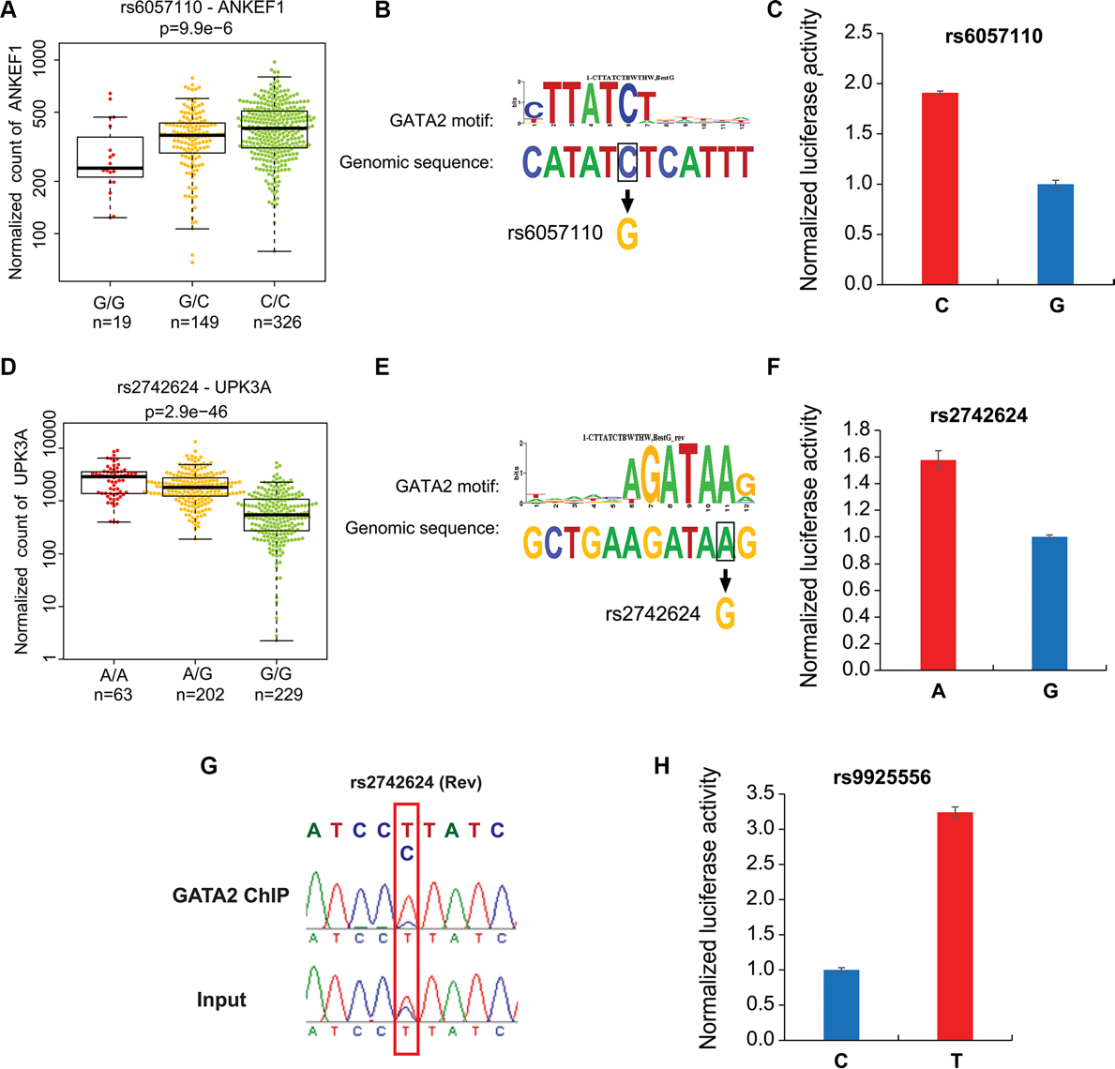
Allele-specific differences in enhancer activity and GATA2 chromatin binding at the selected loci (**A**) The expression of ANKEF1 RNA is shown for samples having homozygous or heterozygous alleles for regulatory SNP rs6057110. Sample size is listed in each genotype. PCa datasets of RNA-seq and SNP array were from TCGA. G allele disrupts a GATA2 motif (**B**) and luciferase reporter assay showed decreased enhancer activity of the G allele at rs6057110 relative to the C allele in LNCaP cells (**C**) (**D**) The expression of UPK3A RNA is shown for patients having homozygous or heterozygous alleles for regulatory SNP rs2742624. G allele disrupts a GATA2 motif (**E**) and decreases enhancer activity compared to the A allele of SNP rs2742624 (**F**) (**G**) GATA2 ChIP was followed by PCR amplification and Sanger sequencing of rs2742624 region. Input sample from ChIP assay was used as a control. Note that rs2742624 is heterozygous in LNCaP cells and a reverse primer (rs2742624_gt_R1, see supplementary Table S3) was used for sequencing. The specific peaks of rs2742624 are highlighted with a red box. Electropherograms showed that GATA2 favors binding to T (reverse strand), corresponding to A allele in reference genomic sequence. (**H**) The enhancer activity for C allele and T allele of rs992556 was determined by luciferase reporter assay.

To confirm that regulatory SNP regions are located in functional enhancers with active histone marks and prostatic TF binding, several genomic regions including rs2659051, rs10936845, rs9925556, rs6057110 and rs2742624 were selected for experimental validation. We performed ChIP-qPCR assays with AR, FOXA1, GATA2, HOXB13 and H3K27ac antibodies or normal IgG as a control. ChIP–qPCR analysis of the positive control gene KLK3 or PSA showed that excessive H3K27ac marker and TFs binding in KLK3 enhancer (Figure 2A, 2G). Overall, all tested SNP regions showed strong enrichment of H3K27ac, at a similar or higher level compared to the KLK3 enhancer (Figure 2A), indicating higher levels of DNA accessibility. Interestingly, our data substantiated striking co-occupancy of 4 TFs in these regions (Figure 2B–2F), suggesting their significance in transcriptional regulation. Alterations to these regulatory regions might result in the disruption of gene expression. Indeed, we observed the strong association between the gene expression level and SNP genotypes (Figures 3A and 3D) and TF binding affinities to DNA motifs were severely affected by SNPs (Figure 3B and 3E).

To further test our hypothesis and to assess whether these enhancers cause allele–specific enhancer activity, we cloned ~160 bp enhancer regions containing individual allele of three SNPs including rs9925556, rs6057110 and rs2742624. Indeed, our luciferase reporter assay data showed dramatic allelic difference of enhancer activity. The enhancer regions with the C allele at rs6057110, A allele at rs2742624 and T allele rs9925556 showed significantly higher (1.5~3 folds) enhancer activity to drive luciferase gene expression in prostate cancer cells, compared to those with another allele respectively (Figure 3C, 3F–3H). These results supported that these regions have allele-dependent enhancer activity, which is highly consistent with genotype-associated gene expression level. Next, we further tested whether the allele-specific enhancer activity is resulted from allele imbalance of TF binding affinity. We chose the rs2742624-containing region because the SNP is heterozygous in LNCaP cells. Indeed, our results showed that the A allele was enriched in anti-GATA2 ChIP-ed DNA fragments compared to input genomic DNA by standard PCR followed by Sanger sequencing analysis (Figure 3G). Thus, GATA2 has binding preference for the A allele of rs2742624, in harmony with higher DNA-binding affinity compared to the G allele (Figure 3E). These observations may explain not only higher luciferase gene expression driven by this A allele-containing enhancer (Figure 3F) but also higher UPK3A expression levels in PCa patients with A/A genotype of this SNP (Figure 3D). Taken together, our results suggested that allele-dependent enhancer activities of these regions are modulated through altering a TF motif /DNA binding affinity.

### The effect of enhancer deletion on target gene expression

**Figure 4:**
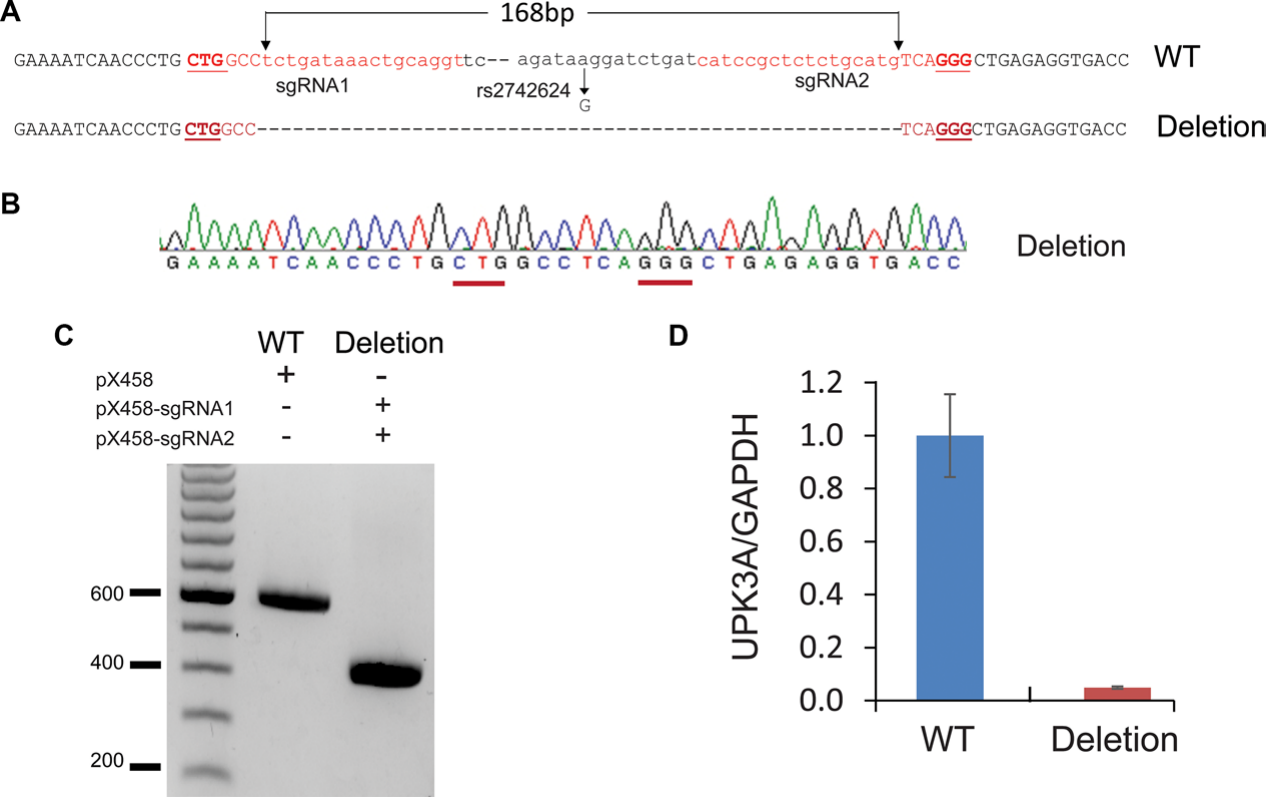
CRISPR/cas9 -mediated genomic deletion of rs2742624 locus diminishes UPK3A gene expression (**A**) The scheme for deletion of rs2742624-containing enhancer by CRISPR/cas9 technology. The guide RNAs were shown in red and protospacer adjacent motifs(PAM) were underlined. (**B**–**C**). PCR genotyping (**C**) and Sanger sequencing (**B**) to confirm the biallelic deletion of the rs2742624 locus (168bp) in the positive clone. (**D**) The expression of UPK3A gene was diminished upon deletion of the rs2742624 locus by RT-qPCR assay, indicating that UPK3A gene is a direct target of the polymorphic enhancer.

Our analyses provide a list of SNPs in PCa-specific functional enhancers that potentially regulate gene expression or their targets. Although luciferase assays can be used to test functional significance of SNP on enhancer activity *in vitro*, the direct evidences that can link enhancer to its specific target gene remain missing. With the advent of CRISP/cas9 genome editing technology [43, 44], now it is possible to delete an enhancer region from the genome and determine changes in gene expression *in vivo*. To examine whether rs2742624-containing enhancer is responsible for UPK3A expression in PCa, we designed a pair of guide RNAs and performed CRISPR/cas9 to delete the target region (Figure 4A–4C). qRT-PCR expression analysis suggested that deletion of the rs2742624 locus resulted in diminished UPK3A level in LNCaP cells, confirming the regulatory role of rs2742624 in UPK3A gene expression (Figure 4D).

## DISCUSSION

Although numerous GWAS studies have been conducted in PCa (reviewed by Broeck et al. [45]), the SNPs detected through GWAS studies are mostly limited to few “Tag SNPs” [46], excluding many other SNPs which are in the LD. These tag SNPs are not necessarily the causative SNPs associated with phenotype. Instead of focusing on GWAS SNPs, we analyzed all individual SNPs resided in prostate-specific regulatory regions to highlight functional variants that modulate TF binding and subsequently affect target gene expression in PCa. Our work thus is complementary to previous GWAS studies. It is also important to note that we focused on active chromatin regions with occupancy of at least one of five prostatic TFs including AR [47], GATA2 [22, 23, 48], FOXA1 [19, 32, 33, 49, 50], HOXB13 [36] and NKX3-1 [20]. Since emerging evidence supported that these TFs are key regulators collaboratively controlling gene expression programs during prostate development, tumorigenesis and/or progression [51], we reasoned that genetic variants located in these regulatory regions can have impact on disease phenotype through affecting expression level of critical target genes. In fact, we identified 38 regulatory SNPs and their target genes in PCa through integrative analysis of genomic, epigenomic and transcriptomic data. Of these SNP-associated genes, 6 have a strong association with biochemical recurrence and 7 are differentially expressed in PCa compared to normal samples. Furthermore, we experimentally confirmed that tested regulatory SNPs influence allelic imbalance of TF binding and allele-dependent enhancer activity, elucidating their mechanistic role in affecting gene expression. However, it needs to be noted that future studies need to be done to determine functional importance of these interesting candidate genes in PCa. It is plausible that other features such as somatic copy-number changes, DNA methylation status and enhancer long-range interactions may be integrated to prioritize putative regulatory SNPs and associated genes, which will be interesting directions for our future studies. Nevertheless, we developed a feasible method to identify significant SNPs and their target genes using publicly available large-scale data sets, bioinformatics tools and experimental validation. The similar procedure could be further tailored to help the identification and validation of functional variants in other diseases.

## MATERIALS AND METHODS

### Publicly available datasets

We used PCa datasets of chromatin features to identify putative regulatory regions. Sequence reads of DNase-seq (GSM816634), ChIP-seq including H3K27ac (GSM1249448), AR (GSM916521), FOXA1 (GSM916523), GATA2 (GSM1600544) and NKX3-1 (GSE28264) were downloaded from the Gene Expression Omnibus database. HOXB13 (PRJEB4865) ChIP-seq data were obtained from the European Nucleotide Archive (http://www.ebi.ac.uk/ena). Sequence reads were aligned to the Human Reference Genome (assembly hg19) using Burrows-Wheeler Alignment (BWA) Tool [30]. Peak calling was performed using HOMER (Hypergeometric Optimization of Motif EnRichment) suite (http://homer.salk.edu/homer/) or MACS [31]. RNA-seq and SNP array of prostate adenocarcinoma were downloaded from the TCGA data portal (https://tcga-data.nci.nih.gov/tcga/). These data include 497 prostate tumor and 52 matched normal samples for RNA-seq and 500 samples for SNP array. The TCGA level3 RNASeqV2 data were generated on the Illumina HiSeq platform, and mapped with the RSEM algorithm and normalized. Genes whose RNA-seq raw counts were all zero across patient samples were removed from list. Affymetrix Genome-wide Human SNP array 6.0 data were processed mainly using the corrected robust linear mixture model (crlmm) algorithm for data normalization and genotype calls.

### Analysis of association between SNPs and nearby gene expression (eQTL)

To identify the SNPs that affect gene expression, RNA-seq and SNP array data from each individual of 494 PCa patients in TCGA data set were collected for analysis. For each SNP, we generated a list of its nearby genes within a 50kb interval (25 kb on either side) using the bedtools and gene annotation file (GRCh37) from Ensembl (http://useast.ensembl.org/Homo_sapiens/). Individuals are grouped according to the allele they carry and expression data of nearby genes are extracted from each individual accordingly. We then applied the Analysis of Variance (ANOVA) test to assess the statistical significance of association between SNP genotypes and expression levels.

### Analysis of SNP-mediated effect on transcription factor binding affinity

The position weight matrix (PWM) models and detection thresholds of transcription factor binding motif for AR, GATA2, FOXA1, HOXB13 and AR, were retrieved from HOMER package. We obtained the genomic sequence +/−20 bp of the given SNP position and then calculated the binding affinity score for each subsequence overlapping the SNP position in either strand. Both the allele-specific scores and between-allele score differences are calculated to evaluate whether the SNP allele impacts the match to PWM significantly, either by disrupting a subsequence with good binding affinity score or creating a subsequence with better score.

### Analysis of association between gene expression and risk of biochemical recurrence

We used a collection of 295 prostate adenocarcinoma samples and 52 normal samples with both RNA-seq gene expression and clinical variables from the TCGA dataset. The gene expression levels are defined as z-scores = (x−μ)/σ, where x is the tumor samples, μ is the mean of normal samples, and σ is the standard deviation of normal samples. Therefore, the scores above and below zero indicate higher and lower expression, respectively. To stratify the tumors into those with high and low gene expression, we first separated them into positive and negative z-scores. We then ranked the tumors based on the z-score in each group. Tumors with high and low gene expression are defined as those in the highest and lowest 75% of tumors with positive and negative z-scores, respectively. Since z-scores close to zero are uninformative and thus not useful in this analysis, we excluded the corresponding tumor samples accordingly. Lastly, we performed Kaplan-Meier analysis using this stratification. Relevant clinical variables, such as relapsed time and status of biochemical recurrence, were extracted from TCGA clinical data file.

### Cell lines and antibodies

LNCaP cells were obtained from American Type Culture Collection and passaged in our laboratory for less than 6 months after resuscitation. LNCaP cells are maintained in RMPI 1640 culture medium supplemented with 10% fetal bovine serum in a 37°C humidified chamber supplemented with 5% CO_2_. Antibodies used in this study include anti- H3K27ac (39133) from Active Motif, anti-FOXA1 (ab23738) from Abcam, anti-AR (sc-815X), anti-GATA2(sc-9008) and anti-HOXB13(sc-66923) from Santa Cruz

### Plasmids and dual luciferase assay

pGL4.26 [luc2/minP/Hygro] vector (Promega) was digested with EcoRV and then blunt-end plasmid DNA was purified and added 3′- T overhang using Taq DNA polymerase to generate pGL4.26-T, which allows direct and high-efficiency TA cloning of PCR products. The selected enhancer regions were amplified by polymerase chain reaction (PCR) using primers listed in Supplementary Table S3 from LNCaP or DU145 genomic DNA using High Fidelity AccuPrime Tag DNA polymerase (Invitrogen) and subcloned into pGL4.26-T, immediately upstream of the minimal promoter (minP) and the luciferase reporter gene. The QuikChange II Site-Directed Mutagenesis kit (Agilent Technologies) was used to obtain either the reference or alternative allele at the SNP site. All clones were confirmed by sequencing. All plasmids were purified from DH5a bacterial cells using the PureYield™ Plasmid Midiprep system (Promega). LNCaP cells were transfected with reporter plasmids along with constitutively active pRL-TK Renilla luciferase plasmid (Promega) using X-tremeGENE HP DNA Transfection Reagent (Roche). At 48 hours after transfection, the cells were lysed with the 1X passive lysis buffer and firefly & Renilla luciferase activities were measured using the Dual-Luciferase Reporter Assay System (Promega) and GloMax^®^−20/20 Single Tube Luminometer (Promega) according to the manufacturer's protocols.

### ChIP

ChIP from LNCaP cells was carried out as described previously [32]. Briefly, cultured cells were crosslinked with 1% formaledehyde for 10 min, and the crosslinking was inactivated by 0.125 M glycine for 5 min at room temperature (RT). Cells were then rinsed with cold 1X PBS twice. The following steps were performed at 4°C: cell pellets were resuspended and incubated in cell lysis buffer + 10 ul/ml PMSF and protease inhibitor (Roche) for 10 min; nuclei pellets were spinned down at 5,000 rpm for 5 min, resuspended in nuclear lysis buffer, and then incubated for another 10 min; chromatin was sonicated to an average length of 500 bp and then centrifuged at 14,000 rmp for 10 min to remove the debris; supernatants containing chromatin fragments were incubated with agarose/protein A or G beads (Upstate) for 15 min and centrifuged at 5,000 rpm for 5 min to reduce nonspecific binding. To immunoprecipitate protein/chromatin complexes, the supernatants were incubated with 3–5 ug of antibody overnight, and then added 50 ul of agarose/protein A or G beads and incubated for 1.5 hour. Beads were washed twice with 1X dialysis buffer and four times with IP wash buffer. The antibody /protein/DNA complexes were eluted with 150 ul IP elution buffer twice. To reverse the crosslinks, the complexes were incubated in elution buffer + 10 ug RNase A and 0.3 M NaCl at 67°C for 4 hours. DNA/proteins were precipitated with ethanol, air-dried, and then dissolved in 100 ul of TE. Proteins were then digested by proteinase K at 45°C for 1 hour, and DNA was purified with QIAGEN PCR column and eluted with 30 ul EB.

### ChIP-qPCR analysis

All primers were designed using Primer 3 (http://biotools.umassmed.edu/bioapps/primer3_www.cgi), and synthesized by Integrated DNA Technologies (Supplementary Table S3). SYBR Green based quantitative real-time PCR was performed using GoTaq qPCR MasterMix (Promega) using a StepOnePlus Real-Time PCR System (Applied Biosystems). ChIP-qPCR enrichment analysis were performed by Comparative Ct method and normalization to input, that is, enrichment over input=2^(−ΔCt)^, where ^Δ^Ct=Ct_sample_-Ct_input._

### Enhancer deletion

CRISPR/Cas9 target sites were identified using the E-CRISPR design tool available at www.e-crisp.org/E-CRISP/, and targets were chosen with the best specificity. To create genomic deletions across the enhancer region of approximately 177–193-bp, two guides RNA were designed to the target sequences Guide RNAs were cloned into BbsI sites of pX458 vector (Addgene plasmid 48138), which encodes both the guide RNA, mammalian Cas9 enzyme with 2A-EGFP. 2 μg total px458 vector (encoding 1 μg each of guides#1 and guide#2) was introduced into LNCaP cells by Neon electroporation system (Life Technologies). Cells were resuspended in 500 uL of RPMI/10% FBS and incubated at 37°C with 5% CO_2_ for 48 hours. BIO-RAD S3™ Cell Sorter was used to capture the cells having high green fluorescent protein signals and then colonies were grown from single cells. Complete deletion of all alleles for a target locus was confirmed by PCR using primers flanking the enhancer. RT-qPCR analysis was performed in triplicate, comparing the deleted cells to parental LNCaP cells.

## SUPPLEMENTARY MATERIALS






