# Supplementary material for: Identification and validation of regulatory SNPs that modulate transcription factor chromatin binding and gene expression in prostate cancer
Source: Oncotarget. 2016 Jul 9;7(34):54616–26. doi: 10.18632/oncotarget.10520 (PMC5338917; doi:10.18632/oncotarget.10520)
Supplement: Supplementary file 1 [file oncotarget-07-54616-s001.pdf]

Identification and validation of regulatory SNPs that modulate transcription factor chromatin binding and gene expression in prostate cancer

Supplementary Materials

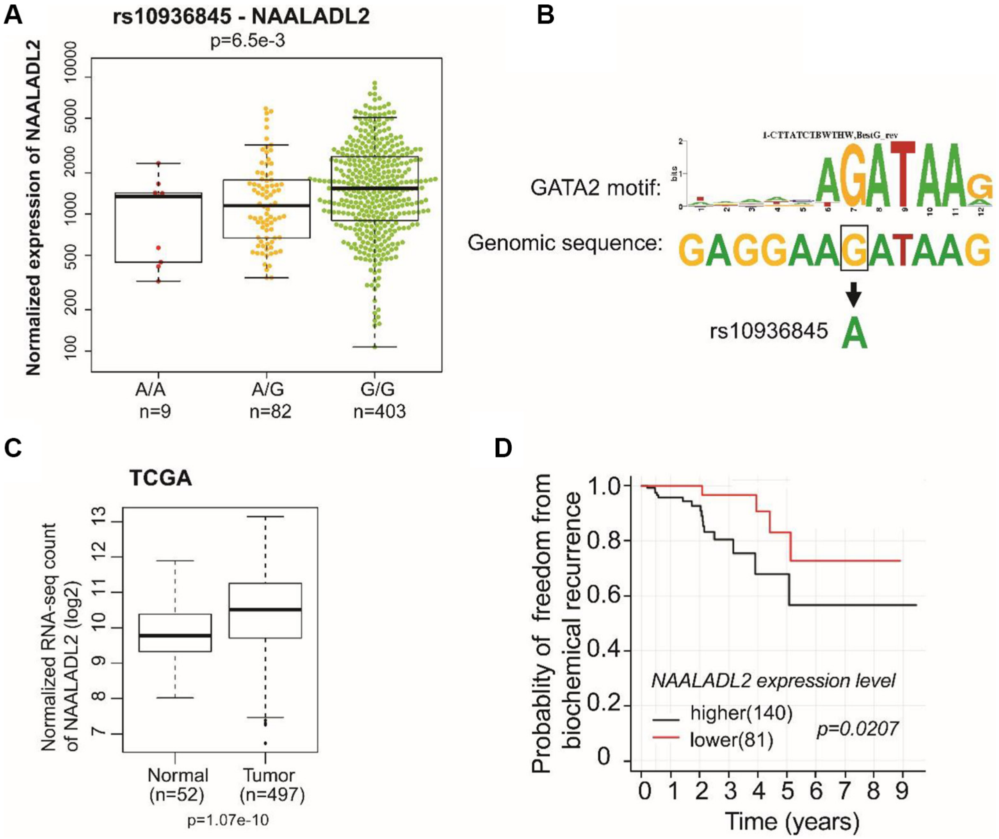

Supplementary Figure S1: Association between rs10936845 genotype, GATA2 motif, NAALADL2 expression and PCa risk. (**A**) The expression of NAALADL2 mRNA in patients is associated with rs6057110 genotypes. *P* value is calculated based on Student's *t*-test. (**B**) The A allele at rs6057110 disrupts a canonical GATA2 motif. (**C**) NAALADL2 mRNA levels in TCGA data set. NAALADL2 is marginally significantly upregulated in PCa compared with benign or normal prostate samples. (**D**) Higher NAALADL2 transcriptional activity is associated with shorter BCR-free survival in TCGA PCa dataset. *P* value is estimated based on the log-rank test.

Supplementary Table S1: SNP-gene pairs from combinatory eQTL and motif analysis. See Supplementary_Table_S1

Supplementary Table S2: Genes showed significantly different expression in tumors relative to normal

SNP ID	Gene	Mean_Normal	Mean_Tumor	*p* value
rs2011345	FMO4	214.7	131.3	1.5346E–11
rs13395911	EFHD1	973.4	410.5	1.0458E–06
rs6760963	MLPH	11171.6	17099.7	2.0094E–13
rs6702939	GIPC2	182.5	60.4	6.662E–09
rs10936845	NAALADL2	879.6	1464.6	1.0745E–10
rs2659051	KLK15	53.5	192.8	9.4027E–09
rs12360799	LYVE1	257.8	56.2	0.00073465

Supplementary Table S3: Oligonucleotides used in this study

Name	sequence (5′->3′)	Application
GAPDH F1	TGCACCACCAACTGCTTAGC	RT-qPCR
GAPDH R1	GGCATGGACTGTGGTCATGAG	RT-qPCR
KLK3 pF1	GCCTGGATCTGAGAGAGATATCATC	ChIP-qPCR
KLK3 pR1	ACACCTTTTTTTTTCTGGATTGTTG	ChIP-qPCR
UPK3A F1	CCATACTCGACGATCGACAC	RT-qPCR
UPK3A R1	TCGTTTCCCCATCAGAACTC	RT-qPCR
rs2742624_gt_F3	TCTGAGCTGGGCCTTTAAGA	genotyping PCR, cloning
rs2742624_gt_R1	CTGCCACAGAAATGCACTGT	genotyping PCR, cloning, sequencing
rs2742624_pF1	GCAGCCCTGTTCAAGAGTTC	ChIP-qPCR
rs2742624_pR1	CCTCTTTGGGTGTCGGTTTA	ChIP-qPCR
rs9925556 pF1	CCACCTCTGCCATTCAAGTC	ChIP-qPCR,cloning,sequencing
rs9925556 pR1	CTCCCGGATGTGCAGTATTT	ChIP-qPCR,cloning
rs6057110_157f	CACTTGTGGGTGAGCTGAAA	ChIP-qPCR, cloning,sequencing
rs6057110_157r	TGCATCTGTGAACTGGCAAT	ChIP-qPCR, cloning
rs2659051 pF1	AGGCTGGGTTTTGTATCCCA	ChIP-qPCR
rs2659051 pR1	TCAGGAGACAGGCAGAAAGG	ChIP-qPCR
rs10936845 pF1	AAGGTGCACAGAGGCAAGTC	ChIP-qPCR
rs10936845 pR1	AGGCAGCCCAATGTAATAGC	ChIP-qPCR
sgRNA_1F	CACCGACCTGCAGTTTATCAGAGGC	guide RNA for CRISPR
sgRNA_1R	AAACGCCTCTGATAAACTGCAGGTC	guide RNA for CRISPR
sgRNA_2F	CACCGCATCCGCTCTCTGCATGTCA	guide RNA for CRISPR
sgRNA_2R	AAACTGACATGCAGAGAGCGGATGC	guide RNA for CRISPR

Supplementary Table S4: Prostate cancer-specific regulatory regions identified in this study. See Supplementary_Table_S4

Supplementary Table S5: All SNPs present in regulatory regions. See Supplementary_Table_S5

Supplementary Table S6: SNP-gene pairs from eQTL analysis. See Supplementary_Table_S6
